# The association between serum uric acid levels, metabolic syndrome and cardiovascular disease in middle aged and elderly Chinese: results from the DYSlipidemia International Study

**DOI:** 10.1186/s12872-015-0059-4

**Published:** 2015-07-11

**Authors:** Yongfeng Tian, Kang Chen, Zongyan Xie, Yuan Fang, Haibin Wang, Yi Nie, Dayi Hu, Yiming Mu

**Affiliations:** Department of Endocrinology, Chinese PLA General Hospital, No.28, Fuxing Rd, Haidian District, Beijing, 100853 P.R. China; Department of Endocrinology, Xinqiao Hospital, Third Military Medical University, No.183, Xinqiao Rd, Shapingba District, Chongqing, 400037 P.R. China; Department of Cardiology, Peking University People’s Hospital, No.11, Xi Zhi Men Nan Da Jie, Xicheng District, Beijing, 100044 P.R. China

**Keywords:** Uric acid, Metabolic syndrome X, Cardiovascular disease, Epidemiology

## Abstract

**Background:**

To explore the association between serum uric acid (SUA) levels, metabolic syndrome (MetS) and cardiovascular disease (CVD) in patients treated with lipid-lowering agents from multiple centers in China.

**Method:**

We investigated 15,478 participants who had been documented with recorded SUA in the DYSlipidemia International Study which included 25,697 patients, aged 45 years old or older, who were treated with lipid-lowering agents from 122 centers between April 2012 and October 2012. Logistic regression analysis was performed to examine the association between SUA levels, MetS and CVD.

**Result:**

After adjusting for multi-variables, hyperuricemia (the highest category of SUA level) showed a significantly higher risk of MetS compared to the lowest category[according to NCEP-ATPIII criteria, odds ratio (OR) 1.51, 95 % confidence interval (CI) (1.30,1.74) in men, OR 2.35 95 % CI (2.00,2.75) in women; and according to IDF criteria, OR 1.40 95 % CI (1.20,1.63) in men, OR 1.65 95 % CI (1.41,1.94) in women]. In addition, elevated SUA concentration was shown to be associated with coronary heart disease (CHD) (OR 1.26 95 % CI (1.09, 1.45) in men, and OR 1.27, 95 % CI (1.07, 1.50) in women) and heart failure (HF) (OR 1.61 95 % CI (1.15, 2.24) in men, and OR 1.91, 95 % CI (1.29, 2.82) in women).

**Conclusion:**

Our research suggested a positive association between SUA levels and MetS in Chinese patients receiving lipid-lowering therapy. Elevated SU levels were positively associated independently of measured confounders to CHD and HF.

## Background

Uric acid is a final enzymatic product from purine degradation in humans. During the past several decades, the prevalence of hyperuricemia appears to be increasing both in western countries and China [1, 2]. There is a growing body of evidence to show that hyperuricemia or elevated serum uric acid (SUA) levels, even within the normal range, are associated with metabolic syndrome (MetS) and its components [3–7]. This relationship is found in children, adolescents and elderly people [8, 9]. Furthermore, whether patients with elevated SUA concentration suffer a higher risk of cardiovascular disease (CVD) is still not conclusive [10–14].

MetS as a CVD risk factor includes the clustering of abdominal obesity, insulin resistance, dyslipidemia, and elevated blood pressure. Insulin resistance is considered to be one of the two central features of patients with MetS [15]. It has been widely demonstrated that MetS is associated with 2-to 3-fold increase in CVD risk and nearly 7-fold for incidence of type 2 diabetes mellitus, (DM) and it also contributes to CVD related mortality and all-cause mortality [16–18].

There are several studies which have reported that there was a positive association between SUA levels and MetS and its components, and also that it was adversely related with elevating CVD risk in Chinese populations. However, few of these studies have explored the direct association between SUA levels and CVD especially in the mainland of China [4, 6, 7, 9, 19]. In addition, the data from subjects from multiple centers in China are still limited. The aim of this present study was to investigate the association between increasing SUA levels, MetS and CVD in participants aged 45 years old or older by using the data from the DYSlipidemia International Study (DYSIS).

## Methods

### Participants and investigators

DYSIS is a series of epidemiologic cross-sectional studies which are performed mainly to assess the blood lipid goal attainment rates in patients receiving lipid-lowering therapy all over the world. 25,697 outpatients from 122 centers in six representative regions across China were included for DYSIS-China between April 2012 and October 2012 [20]. All of the outpatients suffered from dyslipidemia, and received a lipid-lowering therapy for at least 3 months. A total number of 15,478 participants were documented for their SUA level during their visit and were considered for this studied. After excluding 77 subjects who had no data for waist circumference (WC), height or blood pressure, 15,401 participants were eligible for inclusion in our study. All of the participants were aged from 45 to 98 years old. The investigators in our present study were internists, cardiologists, endocrinologists, geriatricians, and neurologists. All the participants provided informed consent to participant in the study. Our study was approved by the Ethics Committee of Peking University People’s Hospital. The approval documents for ethics were regarded as the center of ethical documents for the present study. In addition, informed consent have been provided by all of the participants included in the present study.

### Data collection

Data on socio-demographics (gender, age, and nationality), cardiovascular risk profile (first grade family history of premature CVD, sedentary lifestyle, alcohol consumption, and smoking), cardiovascular history [coronary heart disease(CHD), peripheral arterial disease (PAD), and symptomatic chronic heart failure New York Heart Association(NYHA) class II-IV], and the drugs used for lipid-lowering, DM, hypertension, and antiplatelet were recorded by clinicians at one visit.

Anthropometric data were collected by the clinicians as well. Weight (to the nearest 0.5 Kg) and height (to the nearest 0.5 cm) were measured with light clothes and without shoes. Both systolic and diastolic blood pressures (SBP/DBP) were measured after the participants had rested in the seated position for at least 5 min. WC, also to the nearest 0.5 cm, was measured in the standing position, and the measurement point was the midpoint between the superior border of the iliac crest and the inferior costal margin. Biochemical markers such as triglyceride (TG), total cholesterol (TC), high density lipoprotein cholesterol (HDL-C), low density lipoprotein cholesterol (LDL-C), fasting plasma, glucose (FPG) (documented in 9864 subjects), and SUA were documented at one visit.

### Definition of MetS and CVD

The diagnosis of MetS was attained by using both the definition of the National Cholesterol Education Program Adult Treatment Panel III (NCEP ATP III) [21], and the criteria recommended by the International Diabetes Federation (IDF) [22].According to the NCEP ATP III criteria, participants were considered to have MetS if they had at least three of these five following items: 1) abdominal obesity: WC >102 cm in men and >88 cm in women; 2) TG ≥ 1.70 mmol/L(150 mg/dl); 3) HDL-C <40 mg/dl (1.04 mmol/L) in men and HDL-C <50 mg/dl (1.30 mmol/L) in women; 4) blood pressure: SBP ≥ 130 mmHg, or DBP ≥ 85 mmHg, or previously diagnosed hypertension; 5) fasting plasma glucose: ≥ 5.6 mmol/L (100 mg/ dl), or previously diagnosed DM. While according to the IDF criteria, participants with central obesity (WC > 90 cm in men and >80 cm in women) plus any two of the following criteria were defined as having the MetS: 1) raised triglycerides: ≥ 1.7 mmol/l (150 mg/dl) or specific treatment for this lipid abnormality; 2) reduced HDL-cholesterol: < 1.03 mmol/l (40 mg/dl) in males and < 1.29 mmol/l (50 mg/dl) in females or specific treatment for this lipid abnormality; 3) raised blood pressure: systolic ≥ 130 mmHg or diastolic ≥ 85 mmHg or treatment of previously diagnosed hypertension; 4) raised fasting plasma glucose: fasting plasma glucose ≥ 5.6 mmol/l (100 mg/dl) or previously diagnosed type 2 diabetes (T2DM).

Central obesity was defined as a WC >90 cm in men and >80 cm in women. Hypertension was defined as previously diagnosed hypertension, or patients treated with hypotensors. Hyperuricemia was defined as SUA >420 umol/l in men and >360 umol/l in women. CHD was confirmed as suffering a myocardial infarction or angina, or received a percutaneouse coronary interangioplasty, or coronary artery bypass grafting surgery. Heart failure (HF) was confirmed as NYHA class II-IV. PAD was confirmed as receiving peripheral artery reconstruction surgery.

### Categories and statistical analysis

All statistical analyses were carried out using the SAS 9.2 (SAS Institute Inc., Cary, NC, USA). We classified participants on the basis of sex-specific quartiles of SUA in the normal range and hyperuricemia. The five categories are <280 umol/l, 280–320 umol/l, 320–370 umol/l, 370–420 umol/l, >420 umol/l for males, and <230 umol/l, 230–270 umol/l, 270–310 umol/l, 310–360 umol/l, >360 umol/l for females, respectively. Continuous variables with a normal distribution were presented by mean ± standard deviation. Meanwhile, categorical variables were described by frequency and percentage. Analysis of variance (ANOVA) was performed to access the continuous variables. Chi-square test was then used to calculate the differences between categorical variables. A logistic regression analysis was performed to examine the association between MetS, CVD and the SUA categories of 280–320 umol/l or greater compared to the lowest SUA category of <280 umol/l for men, and the SUA categories of 230–270 umol/l or greater compared to the lowest SUA category of <230 umol/l for women, respectively. Unadjusted odds ratios (OR) and multiple adjusted OR were both calculated. Furthermore, a step-wise multiple linear regression analysis was used to evaluate the association between metabolic risk factors and SUA concentration sex-specifically. At last, we describe the prevalence of MetS according to the sex-specific SUA categories. Statistical significance was set at a probability level of less than 0.05.

## Results

### Clinical characteristics according to serum uric acid levels

The clinical characteristics of the 15,401 eligible participants are summarized according to sex-specific categories of the SUA levels. (Table 1 and Table 2). Participants were older in the highest category of SUA level (hyperuricemia) in women but not in men. Higher SUA levels were significantly adversely associated with weight, body mass index (BMI), WC, SBP, TG, and nonHDL-C, but inversely associated with HDL-C and FPG in both genders (all p values for trend <0.05). Subjects with the highest SUA category exhibited a higher prevalence of hypertension, central obesity, CHD, HF and diuretic agents in both men and women (all p values for trend <0.001). In addition, there existed a higher prevalence of PAD in women with the highest SUA category but not in men. However, there was no significant association between SUA levels and alcohol consumption, family history of premature CVD, TC, LDL-C, and cerebrovascular disease.

**Table 1:** Baseline clinical features of the participants according to serum uric acid level in men

	SUA levels (μmol/L)					*P* value
	<280	280 ~ 320	320 ~ 370	370 ~ 420	>420	
	(*n* = 1748)	(*n* = 1376)	(*n* = 1886)	(*n* = 1452)	(*n* = 1788)	
	Q1	Q2	Q3	Q4	Q5	
Age(years)	66.4(11.09)	65.6(10.80)	65.8(11.19)	65.8(11.17)	66.2(11.48)	0.221
Age ≥ 65 (%)	955(54.6 %)	735(53.4 %)	1004(53.2 %)	774(53.3 %)	964(53.9 %)	0.918
Current smoker (%)	405(23.2 %)	305(22.2 %)	436(23.1 %)	307(21.1 %)	386(21.6 %)	0.015
Alcohol consumption (%)	304(17.4 %)	229(16.6 %)	302(16.0 %)	252(17.4 %)	310(17.3 %)	0.485
Sedentary lifestyle (%)	392(22.4 %)	293(21.3 %)	427(22.6 %)	358(24.7 %)	436(24.4 %)	0.145
Family history of premature CVD (%)	151(8.6 %)	114(8.3 %)	153(8.1 %)	133(9.2 %)	179(10.0 %)	0.282
Height (cm)	169.6(5.73)	170.0(5.54)	170.2(5.78)	169.9(5.52)	169.8(5.83)	0.009
Weight (kg)	70.23(10.30)	70.91(10.06)	71.78(10.10)	72.07(9.94)	72.96(10.85)	<0.001
BMI (kg/m^2^)	24.39(3.11)	24.50(3.00)	24.74(3.04)	24.93(2.96)	25.28(3.33)	<0.001
WC (cm)	88.65(11.08)	89.16(10.63)	89.75(11.36)	90.44(10.57)	91.09(11.15)	<0.001
SBP (mmHg)	130.3(15.25)	129.3(15.41)	130.2(14.67)	130.8(16.16)	131.4(15.71)	0.004
DBP (mmHg)	77.5(9.68)	77.6(9.47)	78.1(9.67)	78.1(9.99)	78.4(10.18)	0.049
FPG (mmol/L)^a^	6.82(2.88)	6.34 (2.27)	6.29(2.28)	6.34(2.35)	6.22(2.11)	<0.001
TC (mmol/L)	4.25(1.13)	4.23 (1.14)	4.22(1.12)	4.27(1.14)	4.28(1.14)	0.577
LDL-C (mmol/L)	2.44 (0.88)	2.43 (0.91)	2.44 (0.94)	2.43 (0.91)	2.42(0.91)	0.924
HDL-C (mmol/L)	1.23(0.35)	1.22 (0.35)	1.17 (0.31)	1.17(0.30)	1.12(0.31)	<0.001
TG (mmol/L)	1.52 (1.21)	1.65(1.32)	1.71 (1.36)	1.87(1.50)	2.18 (1.88)	<0.001
NonHDL-C (mmol/L)	3.01 (1.08)	3.01 (1.09)	3.05(1.07)	3.10(1.09)	3.16(1.08)	0.001
Diabetes mellitus (%)	819(46.9 %)	529(38.4 %)	679(36.0 %)	501(34.5 %)	592(33.1 %)	<0.001
Hypertension (%)	1100(62.9 %)	867(63.0 %)	1286(68.2 %)	1025(70.6 %)	1319(73.8 %)	<0.001
CHD (%)	705(40.3 %)	594(43.2 %)	826(43.8 %)	663(45.7 %)	849(47.5 %)	<0.001
Cerebrovascular disease (%)	384(22.0 %)	256(18.6 %)	379(20.1 %)	284(19.6 %)	335(18.7 %)	0.099
HF (%)	63(3.6 %)	44(3.2 %)	87(4.6 %)	60(4.1 %)	107(6.0 %)	0.001
Peripheral arterial disease (%)	31(1.8 %)	17(1.2 %)	20(1.1 %)	24(1.7 %)	25(1.4 %)	0.378
Antihypertention agents (%)	1012(57.9 %)	827(60.1 %)	1209(64.1 %)	960(66.1 %)	1255(70.2 %)	<0.001
Diuretic agents (%)	52(3.0 %)	33(2.4 %)	77(4.1 %)	76(5.2 %)	139(7.8 %)	<0.001
Antiplatelet agents (%)	1241(71.0 %)	981(71.3 %)	1370(72.6 %)	1057(72.8 %)	1293(72.3 %)	0.710

^a^Among 8250 men, 5211 subjects were documented the FBG. (1107 for Q1, 873 for Q2, 1205 for Q3, 907 for Q4, 1119 for Q5)

Abbreviations: SUA serum uric acid, CVD cardiovascular disease, WC waist circumference, CHD coronary artery disease, SBP systolic blood pressure, DBP diastolic blood pressure, TG triglyceride, TC total cholesterol, HDL-C high density lipoprotein cholesterol, LDL-C low density lipoprotein cholesterol, FPG fasting plasma glucose, HF heart failure, BMI body mass index

**Table 2:** Baseline clinical features of the participants according to serum uric acid level in women

	SUA levels (μmol/L)					*P* value
	<230	230 ~ 270	270 ~ 310	310 ~ 360	>360	
	(*n* = 1359)	(*n* = 1325)	(*n* = 1436)	(*n* = 1389)	(*n* = 1642)	
Age(years)	64.9(10.05)	65.1(9.70)	65.5(9.67)	66.2(9.76)	68.5(9.91)	<0.001
Age ≥ 65 (%)	680(50.0 %)	673(50.8 %)	755(52.6 %)	766(55.1 %)	1058(64.4 %)	<0.001
Current smoker (%)	20(1.5 %)	22(1.7 %)	35(2.4 %)	29(2.1 %)	19(1.2 %)	0.142
Alcohol consumption (%)	7(0.5 %)	7(0.5 %)	13(0.9 %)	7(0.5 %)	9(0.5 %)	0.602
Sedentary lifestyle (%)	228(16.8 %)	233(17.6 %)	257(17.9 %)	279(20.1 %)	336(20.5 %)	0.039
Family history of premature CVD (%)	108(7.9 %)	112(8.5 %)	135(9.4 %)	137(9.9 %)	176(10.7 %)	0.075
Height (cm)	158.1(5.43)	158.3(5.34)	158.3(5.30)	158.1(5.37)	157.7(5.62)	0.045
Weight (kg)	59.92(9.58)	60.64(9.19)	61.36(9.12)	61.85(9.78)	62.52(10.06)	<0.001
BMI (kg/m^2^)	23.92(3.34)	24.19(3.31)	24.46(3.20)	24.72(3.53)	25.09(3.58)	<0.001
WC (cm)	83.34(10.21)	83.69(10.15)	84.36(10.18)	84.62(10.79)	86.38(11.34)	<0.001
SBP (mmHg)	130.1(16.57)	130.0(15.63)	130.2(16.11)	130.5(16.27)	132.2(16.53)	<0.001
DBP (mmHg)	77.1(9.42)	77.4(9.43)	77.0(9.61)	77.1(10.03)	77.1(9.88)	0.889
FPG (mmol/L)^a^	6.62(2.88)	6.35(2.42)	6.21 (2.05)	6.35 (2.25)	6.32 (2.22)	0.007
TC (mmol/L)	4.80 (1.14)	4.83(1.21)	4.85(1.23)	4.78(1.18)	4.79(1.20)	0.504
LDL-C (mmol/L)	2.75 (0.96)	2.76(0.97)	2.76(1.02)	2.76(1.02)	2.76(1.04)	0.964
HDL-C (mmol/L)	1.45(0.39)	1.40(0.37)	1.36 (0.35)	1.32(0.36)	1.26(0.34)	<0.001
TG (mmol/L)	1.63 (1.22)	1.73 (1.08)	1.89 (1.48)	1.98 (1.39)	2.20 (1.55)	<0.001
NonHDL-C (mmol/L)	3.36(1.10)	3.43(1.16)	3.48 (1.20)	3.46(1.13)	3.53 (1.16)	0.001
Diabetes mellitus (%)	509(37.5 %)	443(33.4 %)	485(33.8 %)	521(37.5 %)	669(40.7 %)	<0.001
Hypertension (%)	802(59.0 %)	816(61.6 %)	955(66.5 %)	994(71.6 %)	1270(77.3 %)	<0.001
CHD (%)	389(28.6 %)	411(31.0 %)	473(32.9 %)	496(35.7 %)	661(40.3 %)	<0.001
Cerebrovascular disease (%)	214(15.7 %)	202(15.2 %)	207(14.4 %)	196(14.1 %)	265(16.1 %)	0.493
HF (%)	37(2.7 %)	47(3.5 %)	52(3.6 %)	61(4.4 %)	123(7.5 %)	<0.001
Peripheral arterial disease (%)	8(0.6 %)	9(0.7 %)	19(1.3 %)	15(1.1 %)	26(1.6 %)	0.044
Antihypertention agents (%)	740(54.5 %)	757(57.1 %)	902(62.8 %)	937(67.5 %)	1213(73.9 %)	<0.001
Diuretic agents (%)	44(3.2 %)	59(4.5 %)	78(5.4 %)	80(5.8 %)	159(9.7 %)	<0.001
Antiplatelet agents (%)	769(56.6 %)	765(57.7 %)	861(60.0 %)	868(62.5 %)	1117(68.0 %)	<0.001

^a^Among 7151 women, 4653 subjects were documented the FBG. (852 for Q1, 843 for Q2, 933 for Q3, 911 for Q4, 1114 for Q5)

Abbreviations: SUA serum uric acid, CVD cardiovascular disease, WC waist circumference, CHD coronary artery disease, SBP systolic blood pressure, DBP diastolic blood pressure, TG triglyceride, TC total cholesterol, HDL-C high density lipoprotein cholesterol, LDL-C low density lipoprotein cholesterol, FPG fasting plasma glucose, HF heart failure, BMI body mass index

### The association between serum uric acid categories and MetS

As presented in Table 3 and Table 4, compared with the lowest category of SUA level, the highest category was presented a higher OR for MetS in both genders. Even after multiple adjustments for age, smoking, alcohol consumption, sedentary lifestyle, family history of premature CVD, DM, hypertension, CHD, cerebrovascular disease, HF, PAD, BMI, TC, LDL-C, and diuretic agent use, this association was attenuated but still statistically significant [OR 1.51, 95 % confidence interval (I) (1.30, 1.74) according to NCEP-ATPIII criteria, and OR 1.40 95 % CI (1.20,1.63) according to IDF criteria in men, OR 2.35 95 % CI (2.00, 2.75) according to NCEP-ATPIII criteria, and OR 1.65 95 % CI (1.41, 1.94) according to IDF criteria in women, respectively]. In addition, even in the normal range of, the ORs for MetS were higher with elevated SUA categories only in women. When comparing with the lowest category, according to NCEP ATP III criteria, SUA concentration between 270 ~ 310 μmol/L [OR 1.31, 95 % CI (1.12, 1.54)] and 310 ~ 360 μmol/L [OR 1.62, 95 % CI (1.38, 1.90)] showed a higher risk of prevalence of MetS. Furthermore, we explored the association between SUA level and the components of MetS. When compared with SUV in the lowest category, the highest category of SUA level was significantly associated with central obesity [OR1.29, 95 % CI (1.11, 1.51) in men] and hypertension [OR1.56, 95 % CI (1.34, 1.83) in men, OR 1.66 95 % CI (1.39, 1.98) in women, respectively] after multiple adjustments. In addition, the ORs of hypertension across categories of SUA levels in subjects with normouricemia were 1, 1.07, 1.29, 1.40 (p value for trend < 0.0001) among men, and 1, 1.06, 1.27, 1.50 (p value for trend < 0.0001), respectively.

**Table 3:** The association between SUA and MetS, hypertension, central obesity, and CVD in men

	SUA levels (μmol/L)				
	<280	280 ~ 320	320 ~ 370	370 ~ 420	>420
	Q1	Q2	Q3	Q4	Q5
MetS ATP					
Unadjusted	1(reference)	1.02 (0.88,1.19)	1.16 (1.01,1.34)	1.23 (1.06,1.43)	1.71 (1.49,1.97)
Model 1	1(reference)	1.01 (0.87,1.18)	1.15 (1.00,1.32)	1.22 (1.05,1.42)	1.71 (1.49,1.97)
Model 2	1(reference)	1.01 (0.87,1.18)	1.15 (1.00,1.33)	1.21 (1.04,1.40)	1.71 (1.48,1.96)
Model 3^a^	1(reference)	1.03 (0.88,1.20)	1.16 (1.01,1.34)	1.22 (1.05,1.42)	1.72 (1.49,1.98)
Model 4^1^	1(reference)	1.02 (0.87,1.20)	1.11 (0.96,1.28)	1.13 (0.96,1.31)	1.51 (1.30,1.74)
MetS IDF					
Unadjusted	1(reference)	1.00 (0.86,1.16)	1.20 (1.04,1.37)	1.28 (1.10,1.48)	1.68 (1.46,1.92)
Model 1	1(reference)	0.98 (0.85,1.14)	1.18 (1.03,1.36)	1.26 (1.09,1.46)	1.68 (1.46,1.93)
Model 2	1(reference)	0.99 (0.85,1.15)	1.19 (1.03,1.36)	1.25 (1.08,1.45)	1.67 (1.46,1.92)
Model 3^a^	1(reference)	0.99 (0.85,1.16)	1.19 (1.04,1.37)	1.25 (1.08,1.45)	1.67 (1.46,1.92)
Model 4^1^	1(reference)	0.99 (0.84,1.17)	1.11 (0.96,1.30)	1.13 (0.96,1.32)	1.40 (1.20,1.63)
Central obesity					
Unadjusted	1(reference)	1.11 (0.96,1.27)	1.16 (1.02,1.32)	1.32 (1.15,1.52)	1.60 (1.40,1.82)
Model 1	1(reference)	1.09 (0.95,1.26)	1.15 (1.01,1.31)	1.31 (1.13,1.50)	1.60 (1.40,1.82)
Model 2	1(reference)	1.09 (0.95,1.26)	1.15 (1.01,1.31)	1.29 (1.12,1.49)	1.59 (1.39,1.82)
Model 3^b^	1(reference)	1.12 (0.97,1.30)	1.18(1.03,1.34)	1.32(1.14,1.52)	1.61 (1.41,1.85)
Model 4^2^	1(reference)	1.12 (0.95,1.31)	1.06(0.91,1.23)	1.15(0.98,1.35)	1.29 (1.11,1.51)
Hypertension					
Unadjusted	1(reference)	1.00 (0.87,1.16)	1.26 (1.10,1.45)	1.41 (1.22,1.64)	1.66 (1.44,1.91)
Model 1	1(reference)	1.03 (0.89,1.19)	1.30 (1.13,1.49)	1.46 (1.25,1.69)	1.70 (1.47,1.96)
Model 2	1(reference)	1.03 (0.89,1.20)	1.30 (1.13,1.50)	1.45 (1.24,1.68)	1.70 (1.47,1.96)
Model 3^c^	1(reference)	1.07 (0.92,1.25)	1.35 (1.18,1.560)	1.52 (1.30,1.77)	1.77 (1.53,2.06)
Model 4^3^	1(reference)	1.07 (0.92,1.24)	1.29 (1.12,1.49)	1.40 (1.20,1.64)	1.56 (1.34,1.83)
CHD					
Unadjusted	1(reference)	1.12 (0.97,1.30)	1.15 (1.01,1.32)	1.24 (1.08,1.43)	1.34 (1.17,1.53)
Model 1	1(reference)	1.15 (1.00,1.33)	1.17 (1.03,1.34)	1.27 (1.10,1.46)	1.35 (1.18,1.55)
Model 2	1(reference)	1.14 (0.99,1.32)	1.16 (1.02,1.33)	1.24 (1.07,1.43)	1.33 (1.16,1.52)
Model 3^d^	1(reference)	1.12 (0.97,1.30)	1.11 (0.97,1.28)	1.17 (1.01,1.35)	1.24 (1.08,1.42)
Model 4^4^	1(reference)	1.12 (0.96,1.30)	1.10 (0.96,1.27)	1.18 (1.02,1.37)	1.26 (1.09,1.45)
HF					
Unadjusted	1(reference)	0.88 (0.60,1.31)	1.29 (0.93,1.80)	1.15 (0.80,1.65)	1.70 (1.24,2.34)
Model 1	1(reference)	0.93 (0.63,1.38)	1.34 (0.96,1.86)	1.19 (0.83,1.72)	1.72 (1.25,2.38)
Model 2	1(reference)	0.95 (0.64,1.41)	1.32 (0.94,1.85)	1.16 (0.81,1.68)	1.67 (1.21,2.31)
Model 3^d^	1(reference)	0.96 (0.65,1.43)	1.33 (0.95,1.86)	1.16 (0.81,1.68)	1.65 (1.19,2.29)
Model 4^4^	1(reference)	0.99 (0.66,1.47)	1.30 (0.93,1.83)	1.17 (0.81,1.70)	1.61 (1.15,2.24)
PAD					
Unadjusted	1(reference)	0.69 (0.38 ,1.26)	0.59 (0.34,1.05)	0.93 (0.54,1.59)	0.79 (0.46,1.34)
Model 1	1(reference)	0.72 (0.40,1.31)	0.61 (0.34,1.07)	0.96 (0.56,1.64)	0.79 (0.46,1.34)
Model 2	1(reference)	0.73 (0.40,1.33)	0.59 (0.33,1.04)	0.92 (0.53,1.58)	0.74 (0.43,1.26)
Model 3 ^d^	1(reference)	0.77 (0.42,1.40)	0.62 (0.35,1.10)	0.97 (0.56,1.67)	0.76 (0.45,1.31)
Model 4^4^	1(reference)	0.75 (0.41,1.37)	0.60 (0.34,1.07)	0.93 (0.54,1.61)	0.70 (0.40,1.22)

Model 1: adjusted for age

Model 2: adjusted for variables included in model 1 and sedentary lifestyle, current smoke, alcohol consumption

^a^ Model 3: adjusted for variables included in model 2 and Family history of premature CVD, CHD, cerebrovascular disease, HF, PAD; ^b^ Model 3: adjusted for variables included in model 2 and Family history of premature CVD, CHD, cerebrovascular disease, HF, PAD, T2DM, hypertension; ^c^ Model 3: adjusted for variables included in model 2 and Family history of premature CVD, CHD, cerebrovascular disease, HF, PAD, T2DM; ^d^ Model 3: adjusted for variables included in model 2 and Family history of premature CVD, T2DM, hypertension

^1^ Model 4: adjusted for variables included in model 3^a^ and BMI, TC, LDL-C, Diuretic agents use; ^2^ Model 4: adjusted for variables included in model 3^b^ and BMI, TC, TG, HDL-C, LDL-C, Diuretic agents use; ^3^ Model 4: adjusted for variables included in model 3^C^ and BMI, TC, TG, HDL-C, LDL-C, Diuretic agents use; ^4^ Model 4: adjusted for variables included in model 3^C^ and WC, TC, TG, HDL-C, LDL-C, Diuretic agents use

Abbreviations: SUA serum uric acid, MetS metabolic syndrome, CVD cardiovascular disease, ATP National Cholesterol Education Program Adult Treatment Panel III, IDF International Diabetes Federation, CHD coronary artery disease, TG triglyceride, TC total cholesterol, HDL-C high density lipoprotein cholesterol, LDL-C low density lipoprotein cholesterol, T2DM type 2 diabetes mellitus, HF heart failure, PAD peripheral artery disease

**Table 4:** The association between SUA and MetS, hypertension, central obesity, and CVD in women

	SUA levels (μmol/L)				
	<230	230 ~ 270	270 ~ 310	310 ~ 360	>360
	Q1	Q2	Q3	Q4	Q5
MetS ATP					
Unadjusted	1(reference)	1.13 (0.97,1.33)	1.43 (1.23,1.66)	1.81 (1.55,2.11)	2.77 (2.39,3.21)
Model 1	1(reference)	1.13 (0.97,1.33)	1.43 (1.23,1.66)	1.81 (1.55,2.11)	2.77 (2.39,3.22)
Model 2	1(reference)	1.13 (0.97,1.32)	1.42 (1.22,1.65)	1.79 (1.54,2.09)	2.76 (2.38,3.21)
Model 3^a^	1(reference)	1.12 (0.96,1.32)	1.41 (1.21,1.64)	1.78 (1.53,2.08)	2.71 (2.34,3.16)
Model 4^1^	1(reference)	1.08 (0.92,1.28)	1.31 (1.12,1.54)	1.62 (1.38,1.90)	2.35 (2.00,2.75)
MetS IDF					
Unadjusted	1(reference)	1.01 (0.87,1.17)	1.22 (1.06,1.42)	1.41 (1.21,1.63)	2.05 (1.77,2.37)
Model 1	1(reference)	1.01 (0.87,1.18)	1.23 (1.06,1.42)	1.42 (1.22,1.65)	2.09 (1.80,2.42)
Model 2	1(reference)	1.00 (0.86,1.17)	1.22 (1.05,1.42)	1.40 (1.21,1.63)	2.08 (1.80,2.42)
Model 3^a^	1(reference)	1.00 (0.86,1.17)	1.21 (1.05,1.41)	1.40 (1.20,1.62)	2.06 (1.77,2.39)
Model 4^1^	1(reference)	0.94 (0.80,1.10)	1.08 (0.92,1.26)	1.20 (1.02,1.41)	1.65 (1.41,1.94)
Central obesity					
Unadjusted	1(reference)	1.06 (0.90,1.25)	1.19 (1.01,1.39)	1.26 (1.07,1.48)	1.50 (1.28,1.76)
Model 1	1(reference)	1.06 (0.91,1.25)	1.19 (1.02,1.40)	1.28 (1.08,1.50)	1.56 (1.33,1.83)
Model 2	1(reference)	1.06 (0.90,1.25)	1.19 (1.01,1.40)	1.27 (1.08,1.49)	1.56 (1.33,1.83)
Model 3^b^	1(reference)	1.06 (0.90,1.25)	1.18 (1.00,1.38)	1.24 (1.05,1.46)	1.49 (1.27,1.76)
Model 4^2^	1(reference)	0.95 (0.80,1.14)	0.95 (0.80,1.14)	0.97 (0.80,1.16)	1.04 (0.86,1.25)
Hypertension					
Unadjusted	1(reference)	1.11 (0.95,1.30)	1.38 (1.18,1.61)	1.75 (1.49,2.05)	2.37 (2.02,2.78)
Model 1	1(reference)	1.11 (0.95,1.30)	1.36 (1.16,1.59)	1.68 (1.43,1.98)	2.07 (1.76,2.44)
Model 2	1(reference)	1.10 (0.94,1.29)	1.35 (1.15,1.58)	1.67 (1.42,1.96)	2.06 (1.75,2.43)
Model 3^c^	1(reference)	1.10 (0.94,1.30)	1.36 (1.15,1.60)	1.65 (1.40,1.95)	1.97 (1.67,2.33)
Model 4^3^	1(reference)	1.06 (0.89,1.25)	1.27 (1.08,1.50)	1.50 (1.26,1.79)	1.66 (1.39,1.98)
CHD					
Unadjusted	1(reference)	1.12 (0.95,1.32)	1.22 (1.04,1.44)	1.38 (1.18,1.63)	1.68 (1.44,1.96)
Model 1	1(reference)	1.12 (0.95,1.33)	1.21 (1.02,1.43)	1.32 (1.02,1.43)	1.44 (1.23,1.67)
Model 2	1(reference)	1.12 (0.94,1.33)	1.20 (1.02,1.42)	1.31 (1.11,1.54)	1.42 (1.23,1.67)
Model 3^d^	1(reference)	1.10 (0.93,1.31)	1.15 (0.97,1.36)	1.22 (1.03,1.45)	1.30 (1.11,1.53)
Model 4^4^	1(reference)	1.10 (0.92,1.31)	1.14 (0.96,1.36)	1.20 (1.01,1.42)	1.27 (1.07,1.50)
HF					
Unadjusted	1(reference)	1.31 (0.85,2.04)	1.34 (0.87,2.06)	1.64 (1.08,2.47)	2.89 (1.99,4.21)
Model 1	1(reference)	1.33 (0.86,2.07)	1.33 (0.86,2.04)	1.54 (1.01,2.34)	2.31 (1.58,3.38)
Model 2	1(reference)	1.33 (0.85,2.07)	1.32 (0.85,2.04)	1.48 (0.97,2.26)	2.27 (1.55,3.33)
Model 3^d^	1(reference)	1.35 (0.87,2.11)	1.28 (0.83,1.98)	1.40 (0.92,2.14)	2.13 (1.45,3.12)
Model 4^4^	1(reference)	1.32 (0.85,2.07)	1.22 (0.79,1.90)	1.31 (0.85,2.01)	1.91 (1.29,2.82)
PAD					
Unadjusted	1(reference)	1.15 (0.44,3.00)	2.26 (0.99,5.19)	1.84 (0.78,4.36)	2.72 (1.23,6.02)
Model 1	1(reference)	1.15 (0.44,3.00)	2.24 (0.98,5.13)	1.77 (0.75,4.20)	2.42 (1.09,5.39)
Model 2	1(reference)	1.15 (0.44,3.00)	2.20 (0.96,5.05)	1.68 (0.71,3.99)	2.35 (1.05,5.24)
Model 3^d^	1(reference)	1.20 (0.46,3.13)	2.16 (0.94,4.97)	1.55 (0.65,3.70)	2.09 (0.93,4.68)
Model 4^4^	1(reference)	1.16 (0.44,3.04)	2.10 (0.91,4.87)	1.59 (0.66,3.82)	2.07 (0.91,4.71)

Model 1: adjusted for age

Model 2: adjusted for variables included in model 1 and sedentary lifestyle, current smoke, alcohol consumption

^a^Model 3: adjusted for variables included in model 2 and Family history of premature CVD, CHD, cerebrovascular disease, HF, PAD; ^b^ Model 3: adjusted for variables included in model 2 and Family history of premature CVD, CHD, cerebrovascular disease, HF, PAD, T2DM, hypertension; ^c^ Model 3: adjusted for variables included in model 2 and Family history of premature CVD, CHD, cerebrovascular disease, HF, PAD, T2DM; ^d^ Model 3: adjusted for variables included in model 2 and Family history of premature CVD, T2DM, hypertension

^1^ Model 4: adjusted for variables included in model 3^a^ and BMI, TC, LDL-C, Diuretic agents use; ^2^ Model 4: adjusted for variables included in model 3^b^ and BMI, TC, TG, HDL-C, LDL-C, Diuretic agents use; ^3^ Model 4: adjusted for variables included in model 3^C^ and BMI, TC, TG, HDL-C, LDL-C, Diuretic agents use; ^4^ Model 4: adjusted for variables included in model 3^C^ and WC, TC, TG, HDL-C, LDL-C, Diuretic agents use

Abbreviations: SUA serum uric acid, MetS metabolic syndrome, CVD cardiovascular disease, ATP National Cholesterol Education Program Adult Treatment Panel III, IDF International Diabetes Federation, CHD coronary artery disease, TG triglyceride, TC total cholesterol, HDL-C high density lipoprotein cholesterol, LDL-C low density lipoprotein cholesterol, T2DM type 2 diabetes mellitus, HF heart failure, PAD peripheral artery disease

### The relationship between serum uric acid categories and CVD

The relationship between SUA levels and CVD are also shown in Table 3 and Table 4. In the present study, elevated SUA levels (the highest category) were suggested to be significantly associated with HF and CHD even after multiple adjustments for age, sedentary lifestyle, current smoking habits, alcohol consumption, Family history of premature CVD, T2DM, hypertension WC, TC, TG, HDL-C, LDL-C, diuretic agent use [OR 1.26 95 % CI (1.09,1.45) for CHD, and OR 1.61 95 % CI (1.15,2.24) for HF in men, OR 1.27 95 % CI (1.07,1.50) for CHD, and OR 1.91 95 % CI (1.29,2.82) for HF in women, respectively], when compared to the lowest category SUA level. In addition, no association between SUA level and PAD was found in both men and women after multiple adjustments.

## Discussion

In this study, we investigated the association between SUA levels, MetS and CVD in outpatients who were suffering with dyslipidemia and receiving lipid-lowering therapy from multiple centers in China. Despite several studies which have reported the relationship between SUA level and MetS and elevating CVD risk in Chinese populations from single centers [4, 6, 9], a nationally representative sample is still limited. Furthermore, all of these studies investigated the association between SUA levels and MetS or CVD risk separately, but there was little research on the direct relationship between SUA and CVD in Chinese populations [19]. We found that there was a positive association between SUA levels, MetS and CVD in both men and women.

The association between blood lipid and SUA concentration varies with different participants and sample sizes [4, 6, 8, 9, 23, 24]. Generally, SUA concentration is positively correlated with TG, TC, LDL-C, and negatively correlated with HDL-C. In our study, we also observed that SUA levels were associated with TG and non-HDL-C but inversely associated with HDL-C in outpatients who received a lipid-lowering therapy. Nevertheless, there was no significant relationship between SUA level and TC and LDL-C found in these outpatients. To our knowledge, the underlying biological mechanism between blood lipids and SUA level is still not elucidated. In this present cross-sectional study, statins were used by most of these outpatients, which may be responsible for the unusual relationship between SUA concentration and blood lipids.

An inverse relationship between SUA concentration and FPG was found in the present study and SUA in the lowest category was found to have an extremely high FPG. This negative association of SUA and diabetic parameters was reported in a previous study in Chinese T2DM [4]. Another study in Taiwan also found a negative association between SUA level and hyperglycemia in men, but not in women [23]. Moreover, several studies performed in western populations showed a bell-shaped association between SUA concentration and plasma glucose [25, 26]. Among normal fasting glucose and normal glucose tolerance, a positive association was found between SUA level and FPG and 2-h postprandial glucose (2 h-PPG) [24]. In our study, we found that subjects in the lowest SUA category had a higher prevalence of DM, which is consistent with a previous study [11]. Particularly, in diabetics, hyperglycemia can increase renal excretion of uric acid, leading to decreasing SUA concentration. Furthermore, ethnic differences may also be one reason for the inconsistency between studies. However, we suggest that more prospective cohort studies with different populations should be performed to evaluate the association between SUA concentration and plasma glucose.

Several cross-sectional studies have observed an independent effect of elevating SUA level on MetS [4, 23, 27]. This independent effect is maintained among teenagers and the elderly [8, 9]. Furthermore, longitudinal studies also demonstrated that SUA is an independent predictor for MetS incidence [27, 28]. Similar to those studies, our results showed that elevated SUA level had an independently increased risk of MetS and this was statistically significant between the lowest and highest categories of SUA. Meanwhile, SUA concentration was accompanied with an increasing number of MetS components [4]. This relationship has been shown in both men and women, while the prevalence of MetS was higher among women than among men [3]. A study from Taiwan suggested that the association between SUA level and MetS was more robust in women than in men, in which hyperuricemia was attributed to be a significantly independent predictor for MetS in women, but not in men [29]. Data from another observational study performed in the mainland of China also corresponded to this phenomenon [30]. Similarly, our present study indicated that the positive association remained both genders. Moreover, women seemed to exhibit a stronger association than men.

Several longitudinal studies have indicated that SUA is an independent risk factor of CVD, CVD related mortality and all-cause mortality [10, 11, 31]. However, two well-known cohort studies suggested that SUA levels were not associated with these outcomes [13, 14]. Moreover, several Mendelian randomization Studies, which were performed in westernized populations, suggested that high uric acid or hyperuricemia was causally related to adverse CVD [32, 33]. Controversy about the relationship between SUA levels and CVD has lasted several decades. For Chinese populations, SUA concentration was related with CVD events from two Taiwan cohort studies [19, 34]. Data for this relationship from the mainland of China is still limited. In our present study, we tried to calculate the association between SUA level and CVD in Chinese outpatients treated with lipid-lowering agents. A mild positive association between SUA level and HF and CHD was found in both men and women after adjusting for multi-variables. We suggest that the association between SUA level and CHD and HF may be independent of MetS and other confounders accounted in our present study. However, the causal effect between SUA levels and CHD is still not clear. Moreover, experimental data showed that uric acid may have both anti- and pro-oxidant effects. These two contradictory biochemical reactions may be dependent on the cellular environment [35]. So we suggest more experiments should be performed to make clear the biological mechanism underling the relationship between SUA levels and CVD.

### Limitations

As a cross-sectional study, the present study has some limitations. First, all of the participants enrolled in our study are outpatients who were suffering from dyslipidemia and receiving a lipid-lowering therapy for at least 3 months. Second, although the biochemical markers and CVD events were carefully documented by investigators, there still might be some accidental errors. Confounders, such as plasma insulin, CRP and dietary fructose were not available. Third, the definition of CVD events in our present study varies from previous studies [14, 19]. However, the sample in our present study is large, and all of the participants were from multiple centers in the mainland of China, and the investigators are all clinicians. Furthermore, confounders, such as physical activity, alcohol consumption, the use of diuretics were included in our study. However, further population-based prospective cohort studies should been performed in Chinese populations to confirm the association between SUA, MetS and CVD.

## Conclusions

In summary, serum uric acid was significantly associated with metabolic syndrome, HF and CHD in Chinese outpatients treated with lipid-lowering agents after adjustment for multiple confounders. Moreover, there seems to be an inverse relationship between fasting plasma glucose and serum uric acid. Thus, both experiments in vitro and human trials should be conducted to investigate the biochemical mechanism by which elevated serum uric acid gives rise to metabolic syndrome and CVD.
